# Herpesvirus surveillance in stranded striped dolphins (*Stenella coeruleoalba*) and bottlenose dolphins (*Tursiops truncatus*) from Italy with emphasis on neuropathological characterization

**DOI:** 10.1371/journal.pone.0311767

**Published:** 2024-10-23

**Authors:** Ignacio Vargas-Castro, Federica Giorda, Virginia Mattioda, Maria Goria, Laura Serracca, Katia Varello, Valerio Carta, Sabrina Nodari, Maria Grazia Maniaci, Luana Dell’Atti, Camilla Testori, Nicola Pussini, Barbara Iulini, Roberta Battistini, Simona Zoppi, Fabio Di Nocera, Giuseppe Lucifora, Elena Fontanesi, Pierluigi Acutis, Cristina Casalone, Carla Grattarola, Simone Peletto

**Affiliations:** 1 VISAVET Health Surveillance Centre and Animal Health Department, Veterinary School, Complutense University of Madrid, Madrid, Spain; 2 Istituto Zooprofilattico Sperimentale del Piemonte, Liguria e Valle d’Aosta—WOAH Collaborating Centre for the Health of Marine Mammals, Turin, Italy; 3 Istituto Zooprofilattico Sperimentale del Mezzogiorno, Naples, Italy; 4 Delfini Del Ponente APS, Imperia, Italy; National Cheng Kung University, TAIWAN

## Abstract

Herpesvirus (HV) is widely distributed among cetacean populations, with the highest prevalence reported in the Mediterranean Sea. In this study, a comprehensive analysis was conducted, including epidemiological, phylogenetic, and pathological aspects, with particular emphasis on neuropathology, to better understand the impact of HV in these animals. Our results show a higher presence of HV in males compared to females, with males exhibiting a greater number of positive tissues. Additionally, adults were more frequently affected by HV infection than juveniles, with no infections detected in calves or neonates. The affected species were striped (*Stenella coeruleoalba*) and bottlenose dolphins (*Tursiops truncatus*). The highest positivity rates were observed in the genital system, cerebrum, and skin tissues. Phylogenetic analysis indicated a higher occurrence of Gammaherpesvirus (GHV) sequences but increased genetic diversity within Alphaherpesvirus (AHV). Key neuropathological features included astro-microgliosis (n = 4) and meningitis with minimal to mild perivascular cuffing (n = 2). The presence of concurrent infections with other pathogens, particularly cetacean morbillivirus (CeMV), underscores the complex nature of infectious diseases in cetaceans. However, the presence of lesions at the Central Nervous System (CNS) with molecular positivity for GHV, excluding the involvement of other potential neurotropic agents, would confirm the potential of this HV subfamily to induce neurological damage. Pathological examination identified lesions in other organs that could potentially be associated with HV, characterized by lymphoid depletion and tissue inflammation. These findings enhance our understanding of HV in odontocetes and highlight the need for ongoing research into the factors driving these infections and their broader implications.

## Introduction

Herpesviruses (HV) are enveloped double-stranded DNA viruses that can infect different species of animals, including mammals, birds, reptiles, fish, amphibians, and bivalves [1]. The *Ortoherpesviridae* family comprises three subfamilies: *Alpha*-, *Beta*-, and *Gammaherpesvirinae* [2]. So far, only members of *Alpha*- and *Gammaherpesvirinae* subfamilies have been detected in nine cetacean families, both odontocetes and mysticetes: Delphinidae, Kogiidae, Ziphiidae, Physeteridae, Monodontidae, Phocoenidae, Iniidae and Balaenopteridae [3–11].

These viruses are highly prevalent among stranded cetaceans in different areas of the world, with reported infection rates exceeding 50% [12], 60% [13], 70% [14], and even 80% [15]. However, it is important to note that in some regions, prevalence rates are much lower, often less than 15% [5, 6, 16–19]. This highlights the heterogeneous nature of the data and underscores the need for further studies to understand their impact on cetacean populations. According to the annual stranding reports from the Italian National Reference Centre for Diagnostic Investigations in Stranded Marine Mammals (CReDiMa), the prevalence of HV along the Italian coast between 2016 and 2022 ranged from 10.2–33.3% [20–26]. In 2023, HV prevalence was 15.6% (unpublished data).

HV infections have been associated with a variety of pathological findings in cetaceans, but infections have also been detected without associated pathology [13–15]. Alphaherpesviruses (AHV) have been classically linked to non-suppurative encephalitis [18, 27–29], interstitial nephritis [30], cutaneous and genital lesions [3, 7, 28, 31–33], lymphoid depletion [4], as well as disseminated infections with necrotizing lesions [34]. On the other hand, Gammaherpesvirus (GHV) infections have been mainly associated with cutaneous and mucosal lesions [3, 5, 14, 15, 17, 18, 35–41], and recently, infections have been detected in the central nervous system [9, 14, 15, 29, 42]. However, the neuropathological significance of GHV in cetaceans is still unclear and needs further investigations [14, 15, 29, 42]. Moreover, HV has been associated with immunosuppression [4], which can lead to coinfections with other pathogens [13, 15, 17, 19, 29, 41–43].

Their most characteristic feature is to establish latent infection during which no viral particles are produced [44, 45], and revert to an active replication under stress or immunosuppression [46]. This property may lead to persistent infection interleaved with periodic or continuous shedding [47].

The aim of this study was to investigate the genetic diversity of HV in odontocetes stranded along the Italian coastline between 2016 and 2023, as well as to comprehensively characterize the pathological, and especially neuropathological, findings associated with HV infection in these animals. To achieve this goal, a comprehensive analysis was conducted, including a phylogenetic study, on the sequences detected by CReDiMa during routine diagnosis, to better characterize the different HV strains involved. Additionally, a gross and histopathological examination on positive samples was performed to assess the overall tissue pathology and related lesions. Specifically, to gain a better understanding of the role of HV as a neurotropic agent, the histopathological changes at the level of the Central Nervous System (CNS) were described and scored in the available tissues. The present study contributes to the knowledge of HV in odontocetes and sheds light on the circulation and diversity of this virus along the Italian coasts.

## Materials and methods

### Materials

All animals included in this study were stranded striped dolphins (*Stenella coeruleoalba*) and bottlenose dolphins (*Tursiops truncatus*) submitted to routinely pathological analysis and cause-of-death evaluation by the Italian cetacean stranding network of the Istituti Zooprofilattici Sperimentali (IZS)—veterinary public health institutions under the Italian Ministry of Health and coordinated by CReDiMa. These animals were subjected to a comprehensive post-mortem examination in accordance with established protocols [48, 49].

Epidemiological data including the location and date of stranding, along with biological data such as species, sex, age classification (categorized into newborn-calf, juvenile-subadult, and adult based on total body length) [48, 50], and nutritional and decomposition status, were documented. Carcasses were assessed for decomposition condition (DCC), which was categorized into five codes: extremely fresh (code 1), fresh (code 2), moderate decomposition (code 3), advanced decomposition (code 4), and mummified or skeletal remains (code 5) [49]. The evaluation of nutritional status was assessed morphologically based on anatomical indicators such as the convexity of the dorsal profile, the rib prominence, and blubber thickness measured immediately anterior to the dorsal fin at three locations (dorsal, lateral, and ventral) [49].

During necropsies, representative samples from various organs and lesions were collected and split into three aliquots for subsequent analyses: one was kept frozen at -80°C for biomolecular detection, another was kept in a fridge at 4°C for bacteriological investigations, and the third was preserved in 10% buffered formalin for histological investigations.

When available, ten regions of the CNS were collected and analyzed: basal ganglia, thalamus, midbrain, pons, obex, spinal cord, and frontal, parietal, occipital, and cerebellar cortex. Following fixation in 10% neutral buffered formalin, the tissue specimens were embedded in paraffin, sectioned to 4 ± 2 μm thickness, stained with hematoxylin and eosin (H&E), and observed under a light microscope.

Ancillary investigations included different bacterial, viral, fungal, protozoan and helminthic agents. Main tissue samples, including brain, lung, liver, spleen, and lymph nodes were subjected to standard aerobic, anaerobic, and microaerobic (5% CO_2_) bacterial culture, with subsequent identification through biochemical and/or molecular analyses. Specific bacteriological procedures were applied to samples from different target tissues in order to screen for *Listeria* spp, *Salmonella* spp., and *Brucella* spp [51]. Samples from brain, lung and areas exhibiting macroscopic suspected mycotic lesions or exudates were plated onto Saboraud’s medium for attempted fungal isolation and speciation. Helminth detection involved both macroscopic and microscopic tissue examinations. Endoparasites were conserved in 70% alcohol to facilitate their microscopic identification, according to microscopic morphological features [52, 53].

Biomolecular analyses were performed to detect cetacean morbillivirus (CeMV) [54], *Brucella* [55, 56], *Photobacterium damselae* subsp. *damselae* [57, 58], and *Toxoplasma gondii* [59]. Molecular detection of HV was conducted at CReDiMa using a pan-herpesvirus nested PCR targeting a segment of the DNA polymerase (DNApol) gene, known for its efficacy in identifying novel HV sequences [60]. This approach is also very useful because cetacean HV are generally classified according to the sequence of a part of a locus of their DNApol [61].

The IZS responsible for each stranding case conducted the necropsies and the majority of the ancillary investigations, and CReDiMa supported the other IIZZSS in performing investigations when not set up in the lab in charge.

The results from the post-mortem activities were subsequently retrieved and analyzed by CReDiMa to hypothesize the likely cause of death (COD), in accordance with the pathologist in charge of each case. The CODs were classified into causes of natural origin, including pathologies such as infectious diseases, neonatal/perinatal conditions, traumatic intra-interspecific interactions, senescence/aging, etc., and anthropic origin, such as interaction with fishing activities, ship collisions, etc. This categorization was based on existing literature references [62, 63] and recently developed diagnostic frameworks [64].

For the present study 18 HV-positive dolphins, stranded along the Italian coastline in the period 2016–2023, were selected to perform a phylogenetic and neuropathological characterization, to investigate the genetic diversity of herpesviruses and assess their impact as a neurotropic agent.

A few cases included have also been previously published [42, 65–72].

### Histopathologic CNS characterization

The cerebral cortexes (because always present in the sample set) of the five available positive CNS (ID 2, 6, 7, 10, 12), were re-examined at CReDiMa according to the scheme elaborated by Sierra *et al*. [43] and Giorda *et al*. [42] to comprehensively assess the impact of HV infection on the CNS. The presence and severity of various neuropathological features, such as meningitis, perivascular cuffing, microgliosis, malacia, neuronal necrosis including neuronophagia, intranuclear and/or intracytoplasmatic inclusion bodies (INCIBs), hemorrhages, and edema were evaluated and scored. These lesions were graded on a five-category scale ranging from absent (–) to severe (+ + + +) by two independent pathologists. Furthermore, observations extended beyond the brain cortex, with attention given to nervous system lesions affecting other regions.

### Phylogenetic study

The HV nucleotide sequences were phylogenetically evaluated utilizing the maximum likelihood approach in MEGA 11 software [73]. To ensure the accuracy of sequence alignments and construct a trustworthy phylogenetic tree, we verified the average amino acid p-distance, which was smaller than the acceptance threshold value (< 0.8) for average p-distance [74, 75]. MEGA was used to select the model for maximum likelihood analysis, determining that the Tamura 3-parameter model with a discrete Gamma distribution to model evolutionary rate differences among sites with five categories was the most suitable. Additionally, 2000 bootstrap replicates were employed. The resulting tree was rooted and edited using iTOL editor [76].

Based on a prior BLAST analysis, this phylogenetic study included one sequence of human herpesvirus, one sequence of bat herpesvirus, and 27 sequences of cetacean herpesvirus detected in different countries worldwide. Furthermore, the sequences documented in this study were integrated.

### Inclusivity in global research

Additional information regarding the ethical, cultural, and scientific considerations specific to inclusivity in global research is included in the S1 Checklist.

### Ethics approval and consent to participate

Ethical review and approval was not required for the animal study because in the collection of post-mortem tissues for research purposes, the approval of the corresponding Ethics Committee is not required.

## Results

Between 2016 and 2023, 18 dolphins tested positive for HV, and 19 sequences were identified and deposited in GenBank under accession numbers **PP505967**-**PP505985**.

The biological and epidemiological data from the analyzed individuals are summarized in **Table 1**. Among the 18 HV-positive cetaceans included in this study, 12 were males, five were females, and in one case, the sex could not be determined due to the advanced decomposition of the carcass. Concerning age distribution, 10 individuals were classified as adults, while eight were categorized as juveniles-subadults. Notably, no calves or neonates were found within the HV-positive sample. In terms of species, HV was detected in 15 striped dolphins and three bottlenose dolphins. Regarding the geographical locations of the strandings of affected dolphins, 13 were found in Liguria, three in Campania, and two in Calabria regions.

**Table 1:** Selected Herpesvirus-infected cetaceans stranded along the Italian coastline between 2016 and 2023, arranged in chronological order, with the corresponding CReDiMa laboratory reference. For each individual are detailed informations on individual data (decomposition condition category-DCC, nutritional condition category-NCC, species, age, sex, stranding date, stranding location and sea sector), tissues tested for Herpesvirus molecular analysis, coinfections and the likely cause of death (COD). Legend: positive Herpesvirus-infected tissues are underlined. GenBank accession numbers of HV sequences are indicated in parentheses. Abbreviations: ID, case identification; Ref. lab, C.Re.Di.Ma. Laboratory reference; DCC, decomposition condition category; NCC, nutritional condition category; SD, Stranding Date (dd/mm/yyyy); COD, likely cause of death; J, juvenile-subadult; A, adult; M, male; F, female; ND, Not determined; NA, not available; Long, longitude; Lat, latitude; AHV, Alphaherpesvirus; GHV, Gammaherpesvirus. Since some of the animals were previously included in other works, primarily associated with different agents, references to these publications have been provided.

ID	Ref. lab	DCC	NCC	Species	Age	Sex	SD	Region (Sea Sector)	Stranding Location	Tissues tested	Coinfections	Suggestive causative agent of pathological features	COD (classification, origin, sub-category)	References
1	20250	2	Moderate	*Stenella coeruleoalba*	J	F	04/03/2016	Liguria (Ligurian Sea)	Long: 8:10.121 Lat: 44:0.065	Tracheobronchial lymph node, prescapular lymph node, lung (AHV, **PP505973**), spleen, kidney, cerebrum, urinary bladder, thymus, mesenteric lymph node	*Aspergillus fumigatus* *Staphylococcus* spp *E*. *coli*	HV *A*. *fumigatus*	*Classification*: natural *Origin*: infectious diseases *Sub-category*: coinfection (viral–mycotic)	[65]
2	95842	2	Poor	*Stenella coeruleoalba*	J	M	20/11/2016	Liguria (Ligurian Sea)	Long: 8:58.965 Lat: 44:23.509	Cerebrum (GHV, **PP505967**), skin lesions, spleen, lung, mediastinic and prescapular lymph nodes	CeMV *Photobacterium damselae*	CeMV HV	*Classification*: natural *Origin*: infectious diseases *Sub-category*: viral	[42, 66, 67]
3	78983	2	Poor	*Stenella coeruleoalba*	A	F	14/09/2017	Liguria (Ligurian Sea)	Long: 8:27.27 Lat: 44:17.437	Prescapular lymph node (AHV, **PP505976**), cerebrum, spleen, kidney, tracheobronchial lymph node	Monophasic variant of *Salmonella typhimurium*, CeMV, *Toxoplasma gondii*, *Photobacterium damselae*	*S*. *typhimurium* CeMV HV *T*. *gondii*	*Classification*: natural *Origin*: infectious diseases *Sub-category*: coinfection (bacterial–viral–parasitic)	[42, 68]
4	2926	2	Moderate	*Stenella coeruleoalba*	J	M	13/01/2018	Liguria (Ligurian Sea)	Long: 8:1.083 Lat: 43:52.437	Tracheobronchial lymph node (AHV, **PP505977**), prescapular lymph node, spleen, lung, cerebrum, skin lesions	CeMV *Photobacterium damselae*	CeMV HV	*Classification*: natural *Origin*: infectious diseases *Sub-category*: viral	[42, 69]
5	5129	4	Good	*Stenella coeruleoalba*	A	M	20/01/2018	Liguria (Ligurian Sea)	Long: 8:27.089 Lat: 44:15.904	Pulmonary lymph node (AHV, **PP505978**), liver, lung, prescapular lymph node, spleen, cerebrum	Monophasic variant of *Salmonella typhimurium*, CeMV *Photobacterium damselae*	CeMV HV *S*. *typhimurium*	*Classification*: natural *Origin*: infectious diseases *Sub-category*: coinfection (viral -bacterial)	[68, 69]
6	5386	1	Poor	*Stenella coeruleoalba*	A	M	21/01/2018	Liguria (Ligurian Sea)	Long: 8:46.629 Lat: 44:25.579	Genital lesion (GHV, **PP505968**), lingual ulcer, cerebrum, prescapular lymph node; lung, tracheobronchial lymph node, pulmonary lymph node, spleen, kidney	CeMV *Salmonella Tsevie*, *Photobacterium damselae*	CeMV HV	*Classification*: natural *Origin*: infectious diseases *Sub-category*: viral	
7	62728	2	Poor	*Stenella coeruleoalba*	A	M	26/06/2018	Calabria (Southern Tyrrhenian Sea)	Long: 16:6.393 Lat: 38:42.698	Cerebrum (GHV, **PP505970**)	CeMV *Toxoplasma gondii* *Vibrio* spp	CeMV *T*. *gondii* HV	*Classification*: natural *Origin*: infectious diseases *Sub-category*: coinfection (viral-parasitic)	[42, 69, 70]
8	18013	3	Good	*Tursiops truncatus*	J	M	23/02/2019	Liguria (Ligurian Sea)	Long: 8:4.951 Lat: 43:54.42	Lung (AHV, **PP505974**), prescapular, tracheobronchial and pulmonary lymph nodes, spleen, kidney, cerebrum, skin lesion	*Enterococcus faecium Photobacterium damselae*, *Staphylococcus* spp, Mucorales		*Classification*: ND	[71]
9	62877	3	Moderate	*Stenella coeruleoalba*	A	M	21/07/2019	Liguria (Ligurian Sea)	Long: 8:9.449 Lat: 43:56.975	Cerebrum (AHV, **PP505975**), lung, prescapular, pulmonary, mesenteric lymph nodes, spleen, kidney	CeMV *Photobacterium damselae*	CeMV *P*. *damselae*	*Classification*: natural *Origin*: infectious diseases *Sub-category*: coinfection (viral-bacterial)	[69]
10	12046	2	Poor	*Stenella coeruleoalba*	J	M	23/12/2019	Campania (Central Tyrrhenian Sea)	Long: 14:7.924 Lat: 40:49.224	Cerebrum (AHV, **PP505971**)	Cetacean morbillivirus, *Photobacterium damselae*	CeMV *P*. *damselae*	*Classification*: natural *Origin*: infectious diseases *Sub-category*: coinfection (viral-bacterial)	
11	6128	2	Good	*Stenella coeruleoalba*	A	M	22/01/2021	Liguria (Ligurian Sea)	Long: 9:13.45 Lat: 44:20.416	Kidney (GHV, **PP505979**), spleen, prescapular and pulmonary lymph nodes, lung, cerebrum	-		*Classification*: ND	
12	24621	2	Good	*Stenella coeruleoalba*	A	M	11/02/2021	Campania (Central Tyrrhenian Sea)	Long: 14:2.848 Lat: 40:49.463	Cerebrum (AHV, **PP505972**), kidney, skin, spleen, prescapular lymph node	CeMV *T*. *gondii* *B*. *ceti*	CeMV *T*. *gondii* HV *B*. *ceti*	*Classification*: natural *Origin*: infectious diseases *Sub-category*: coinfection (viral-bacterial-parasitic)	[70, 72]
13	24628	2	Good	*Stenella coeruleoalba*	A	F	18/02/2021	Campania (Central Tyrrhenian Sea)	Long: 14:2.779 Lat: 40:48.941	Lung (GHV, **PP505969**), skin (GHV, **PP505980**), cerebrum, spleen, kidney, prescapular lymph node	CeMV- *B*. *ceti*	*B*. *ceti* CeMV	*Classification*: natural *Origin*: infectious diseases *Sub-category*: coinfection (bacterial-viral)	[70, 72]
14	177	2	Good	*Tursiops truncatus*	A	F	24/12/2021	Liguria (Ligurian Sea)	Long: 8:27.233 Lat: 44:17.417	Skin lesions (GHV, **PP505981**), lung, prescapular and mesenteric lymph nodes, spleen, kidney	CeMV	CeMV HV	*Classification*: natural *Origin*: infectious diseases *Sub-category*: viral	[69, 71]
15	8319	1	Moderate	*Stenella coeruleoalba*	J	M	30/01/2022	Liguria (Ligurian Sea)	NA	Pulmonary lymph node (GHV, **PP505982**), prescapular lymph node, lung, kidney, cerebrum, skin lesions	*Photobacterium damselae*, *Clostridium perfringens*	HV *C*. *perfringens* *P*. *damseale*	*Classification*: natural *Origin*: infectious diseases *Sub-category*: coinfection (viral-bacterial)	
16	6054	2	Moderate	*Stenella coeruleoalba*	J	F	21/01/2023	Liguria (Ligurian Sea)	NA	Lung (GHV, **PP505983**), pulmonary lymph node, prescapular lymph node, spleen, kidney, skin lesions	*Photobacterium damselae*, *Staphylococcus haemolyticus*, *Clostridium perfringens*	*S*. *haemolyticus*	*Classification*: natural *Origin*: infectious diseases *Sub-category*: bacterial	
17	84796	2	Moderate	*Stenella coeruleoalba*	J	M	04/07/2023	Calabria (Ionian Sea)	NA	Spleen (GHV, **PP505985**), cerebrum, lymph nodes pool, kidney, lung, skin	-	*-*	*Classification*: ND	
18	88778	4	ND	*Tursiops truncatus*	A	ND	10/10/2023	Liguria (Ligurian Sea)	NA	Mesenteric lymph node (GHV, **PP505984**), lung, spleen, kidney	-	*-*	*Classification*: ND	

The tissues in which HV was most frequently detected were lymph nodes (n = 14), cerebrum (n = 7), lung (n = 5). However, the organ systems that had the highest percentages of HV-positive samples (100 * HV-positive samples/number of samples evaluated) were genital (1/1, 100%), nervous (7/14, 50.0%), integumentary (4/8, 50.0%), and hematopoietic (14/31, 45.16%).

Co-infections were detected in 15 animals (**Table 1**), involving bacteria (n = 12), viruses (n = 11), protozoa (n = 3), helminths (n = 3), and fungi (n = 2). The predominant agent identified in these coinfections was CeMV (n = 11).

**Fig 1:**
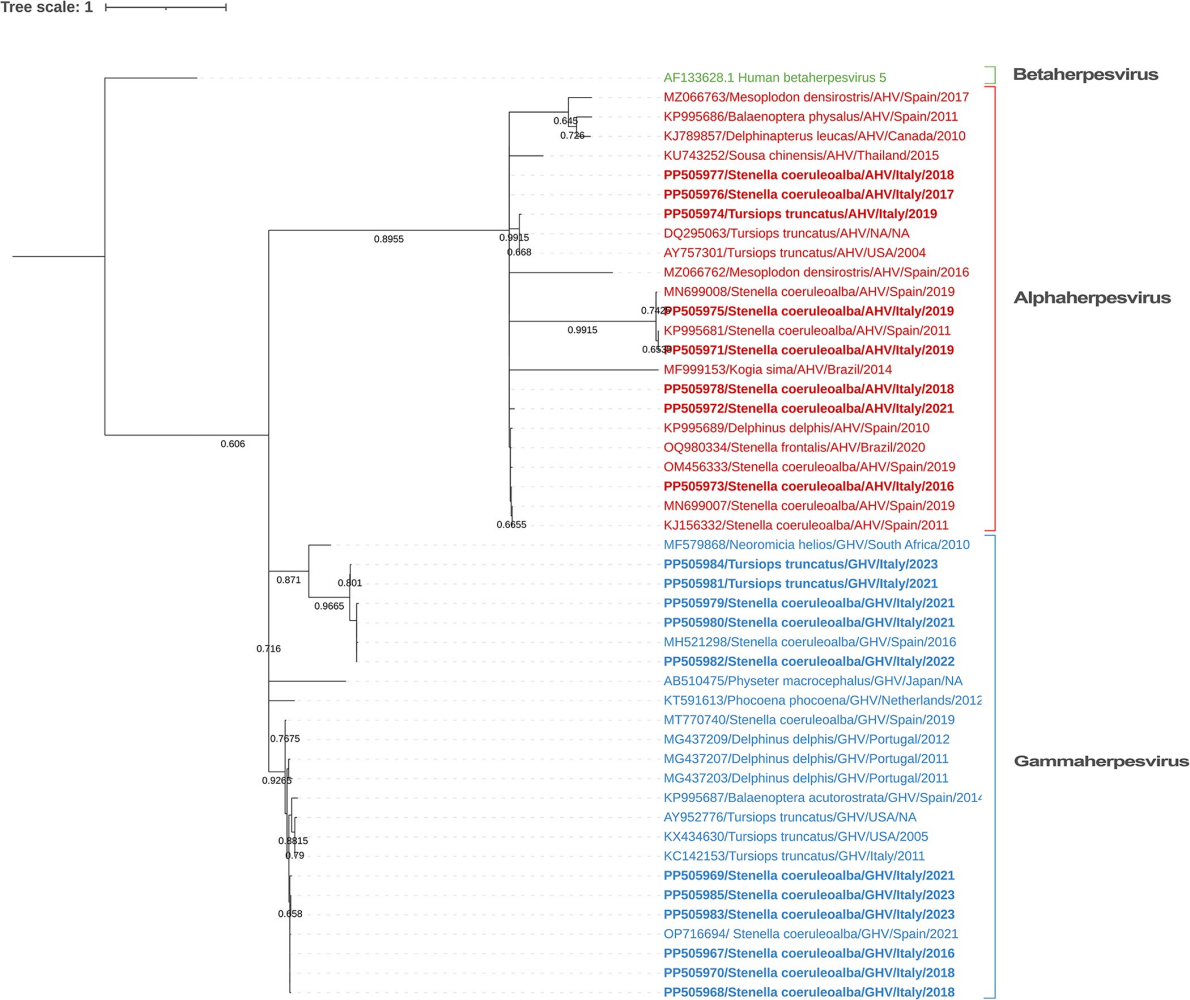
Maximum-likelihood phylogram of herpesviruses based on partial DNApol gene sequences. Sequences are labeled with accession numbers, host species, herpesvirus subfamily, and country and year of origin. The nucleotide sequences reported in the present study are highlighted in bold.

The constructed phylogeny unveiled two significant branches separating GHV and AHV (**Fig 1**). Within the GHV branch the sequences described in this study form a cluster (accession numbers **PP505979**—**PP505982**, **PP505984**) along with another sequence previously documented from a striped dolphin stranded on the Spanish Mediterranean Coast (although unpublished, the sequence is available in GenBank, under accession number **MH521298**), distinct from the rest of the GHV sequences. Moreover, there is another sequence (accession number **MF579868**), more distantly related phylogenetically, yet aligning with the cluster as an outgroup, identified in a bat species (*Neoromicia helios*).

The phylogenetic analysis revealed that eight strains of AHV and eleven strains of GHV were identified. Noteworthy, one animal (ID 13) exhibited the presence of two distinct GHV, one in the lung (accession number **PP505969**) and the other in the skin (accession number **PP505980**).

The macroscopic lesions observed in HV-positive animals are summarized in **Table 2**, encompassing all significant changes that are potentially associated with HV. Conversely, histopathology was performed on 13 out of 18 (72.2%) HV-positive dolphins, focusing solely on the organs showing positive results. The microscopic lesions identified in HV-positive tissues are summarized in **Table 2**, emphasizing neuropathological changes such as astro-microgliosis (n = 5) and meningitis with minimal to mild perivascular cuffing (n = 3), lymphoid depletion and inflammation of the affected tissues.

**Table 2:** Gross and microscopic pathologic findings in HV-positive tissues of striped dolphins and bottlenose dolphins included in this study. Macro- and/or microscopic lesions potentially related to HV are underlineated. Abbreviations: ID, case identification; NSLO, no significant lesions observed; NS, non suppurative. Since some of the animals were previously included in other works, primarily associated with different agents, references to these publications have been provided.

ID	Macroscopic lesions	Microscopic lesions	References
1	Thoracic reactive lymphadenopathy	**Lung**: multifocal pyogranulomatous bronchopneumonia with alveolar flooding of oedema and haemorrhage throughout the pulmonary parenchyma. **Spleen**: lymphoid hyperplasia, with follicular hyperplasia and sinus hypercellularity with plasma cells, macrophages and rare eosinophils **Prescapular and tracheobronchial lymph nodes**: lymphoid hyperplasia, with sinus hypercellularity with plasma cells, macrophages, eosinophils, rare neutrophils and scattered haemorrhagic foci	[65]
2	Multifocal skin erosions (rostrum, left flank, caudal peduncle) (**Fig 4**)	**CNS**: astro-microgliosis and spongiotic changes in white matter associated to mild NS plexus choroidits (**Table 3** and **Fig 2A and 2B**). **Lesion 1**: hyperplastic dermatitis with parasitic granuloma, with presence of parasitic structures surrounded by lymphohistiocytic reaction and thick fibrous capsule. **Lesion 2**: degenerative and necrotizing dermatitis with areas of epithelial erosion and necrosis, and marked hyperemia at the dermoepidermal junction, with the presence of a mononuclear inflammatory infiltrate. **Lesion 3**: degenerative and necrotizing dermatitis with area of epithelial loss, moderate mononuclear inflammatory infiltrate in the dermis and mild vascular dilatation.	[42, 66, 67]
3	Multicentric reactive lymphadenopathy	**Prescapular lymph node**: lymphoid depletion (**Fig 7**)	[42, 68]
4	Prescapular lymphadenopathy and congestive splenomegaly	**Prescapular lymph node**: moderate lymphoid depletion with hyperplasia and hypertrophy of residual follicles. Marked hypercellularity of sinuses, characterized by histiocytes, plasma cells, lymphocytes, and rare degenerated neutrophils. **Tracheobronchial lymph node**: moderate lymphoid depletion with hyperplasia and hypertrophy of residual follicles. Hypercellularity of sinuses characterized by histiocytes, plasma cells, and eosinophilic. Marked vascular congestion. **Spleen**: lymphoid hyperplasia, with follicular hyperplasia and sinus hypercellularity, with lymphocytes, plasma cells, and numerous cells with cariorrhexis. **Lung** (caudal portion): moderate multifocal broncho-interstitial pneumonia with marked dilatation of alveolar and vascular capillaries, alveolar emphysema, with alveolar rupture and alveolar walls multifocally infiltrated by mononuclear inflammatory cells, and bronchiolar wall muscles thickened with eroded epithelium. Additionally, multiple granulomatous lesions with central mineralization, likely of parasitic origin.	[42, 69]
5	Not available (generalized post-mortem autolysis)	Not available	[68, 69]
6	Ulcerative glossitis (**Fig 6**) and esophagitis genital caseous exudate, proliferative balanitis (**Fig 5**), with a whitish proliferative structure of 2 cm in length; generalized lymphadenopathy, splenomegaly with petechiae; edematous prescapular lymphnodes	**Lingual ulcer**: glossitis, with epithelial hyperplasia with formation of islands and cords of cells in the submucosa. Mitotic figures at the basal layer and balloon-like degeneration of epithelial cells in the superficial layers. Mild mixed inflammation in the submucosa. **Prescapular lymph node**: moderate lymphoid depletion with hyperplasia and hypertrophy of residual follicles showing hyalinosis and centrofollicular histiocytic infiltration. Additionally, hypercellularity of sinuses mainly composed of histiocytes with rare predominantly eosinophilic polymorphonuclear cells. **Genital lesion**: hyperplastic balanitis with esophytic lesion characterized by hyperplasia of the epithelium with extension of islands and cords of cells of the underlying dermis. Presence of mitotic figures and melanin pigment in the deep layer, extending to even the most superficial layers. Ballooning degeneration of epithelial cells at the level of the stratum corneum. **CNS**: mild NS meningoencephalitis (**Table 3**)	
7	Meningeal congestion and hemorrhages.	CNS: mild non-suppurative meningoencephalitis (**Table 3** and **Fig 2C)**	[42, 69, 70]
8	Parasitic pneumonia, with scattered subpleural and parenchymal calcified granulomas	Not available	[71]
9	Not available (internal organs and central nervous system in an advanced state of autolysis)	Not available	[69]
10	Meningeal and cerebral congestion	**CNS**: mild pyogranulomatous plexus choroiditis (**Table 3** and **Fig 2D** and **2E**)	
11	Spleen, kidney: NSLO	**Spleen**: lymphoid depletion. Diffuse congestion, with blood stasis and dilated blood vessels engorged with erythrocytes. **Kidney**: diffuse congestion. with blood stasis in glomerular and interstitial capillaries, and dilated blood vessels engorged with erythrocytes.	
12	Hyperemic meninges, brain and cerebellum	**CNS**: Severe NS meningoencephalitis associated to plexus choroiditis (**Table 3** and **Fig 2F**). **Kidney**: lymphoplasmacytis nephritis with diffuse congestion.	
13	Haemorrhagic pneumonia; bronchopneumonia by nematodes	**Lung:** congestion and acute neutrophilic and histiocytis bronchointerstitial pneumonia	
14	Multifocal skin erosions	**Skin lesions:** severe pyogranulomatous pannicultis	
15	Prescapular lymphadenopathy with a congested-hemorrhagic appearance	**Prescapular lymph node**: moderate diffuse lymphoid depletion with sinus histiocytosis, presence of red blood cells and extensive parenchymal hemorrhagic areas. **Pulmonary lymph node**: moderate diffuse lymphoid depletion with sinus histiocytosis, presence of red blood cells and parenchymal hemorrhagic areas. Hemorrhagic areas are also observed in the surrounding adipose tissue. **Lung**: moderate multifocal broncho-interstitial pneumonia with diffuse dilatation of alveolar and vascular capillaries due to congestion. And rare hemorrhagic foci. Additionally, rare foci of lymphoplasmacytic and eosinophilic inflammatory infiltrate. Pulmonary parenchyma with moderate multifocal fibroplasia and marked dilatation of alveolar capillaries with presence of mononuclear inflammatory infiltrate in inter-alveolar septa. .	
16	Diffuse pulmonary emphysema	**Lung**: diffuse congestion, with locally extensive hemorrhages emphysema and locally alveolar wall destruction	
17	Spleen: NSLO	Not available	
18	Not available (internal organs and central nervous system in an advanced state of autolysis)	Not available	

**Fig 2:**
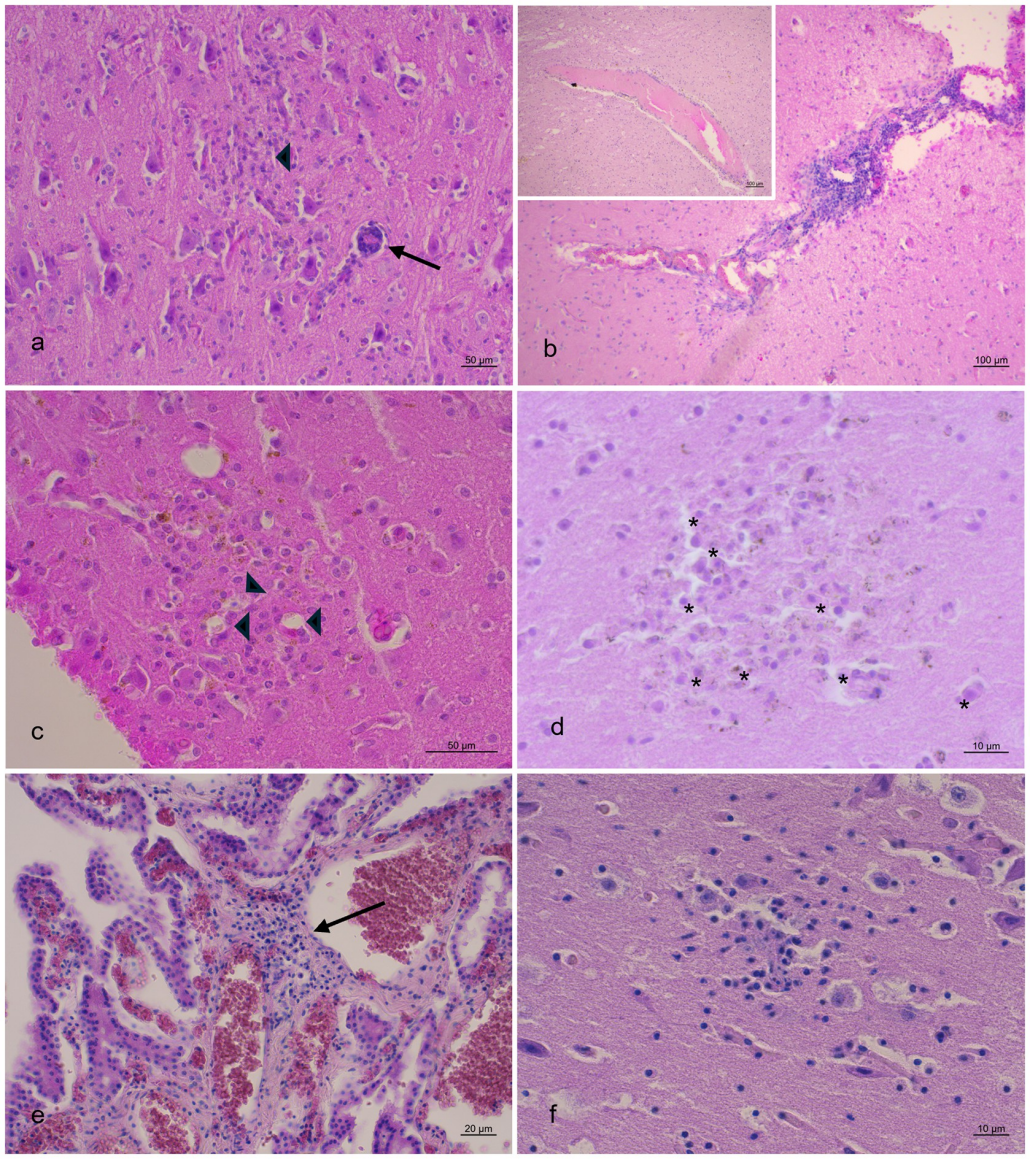
Neuropathological changes in stranded cetaceans affected by Herpesvirus. Animals are identified using the case identification provided in **Table 1** (ID). (A) Parietal cortex (ID6). Area of gliosis and neuronophagia (arrowhead) and perivascular cuffings characterized by mononuclear cell infiltrates (arrowhead). HE. (B) Parietal cortex (ID6). Moderate non-suppurative meningitis. HE. Inset: Parietal cortex. Lymphoplasmacytic perivascular cuff of a congested vessel. HE. (C) Occipital Cortex (ID6). Area of gliosis and neuronophagia with neuronal loss (arrowheads). HE. (D) Frontal cortex (ID7). Malacic area with several gitter cells (asterisks). HE. (E) Plexus choroideus (ID 10). Focal and mild pyogranulomatous plexus choroiditis (arrow). HE. (F) Parietal cortex (ID10). Neuronophagic nodule of glia cells. HE.

**Fig 3:**
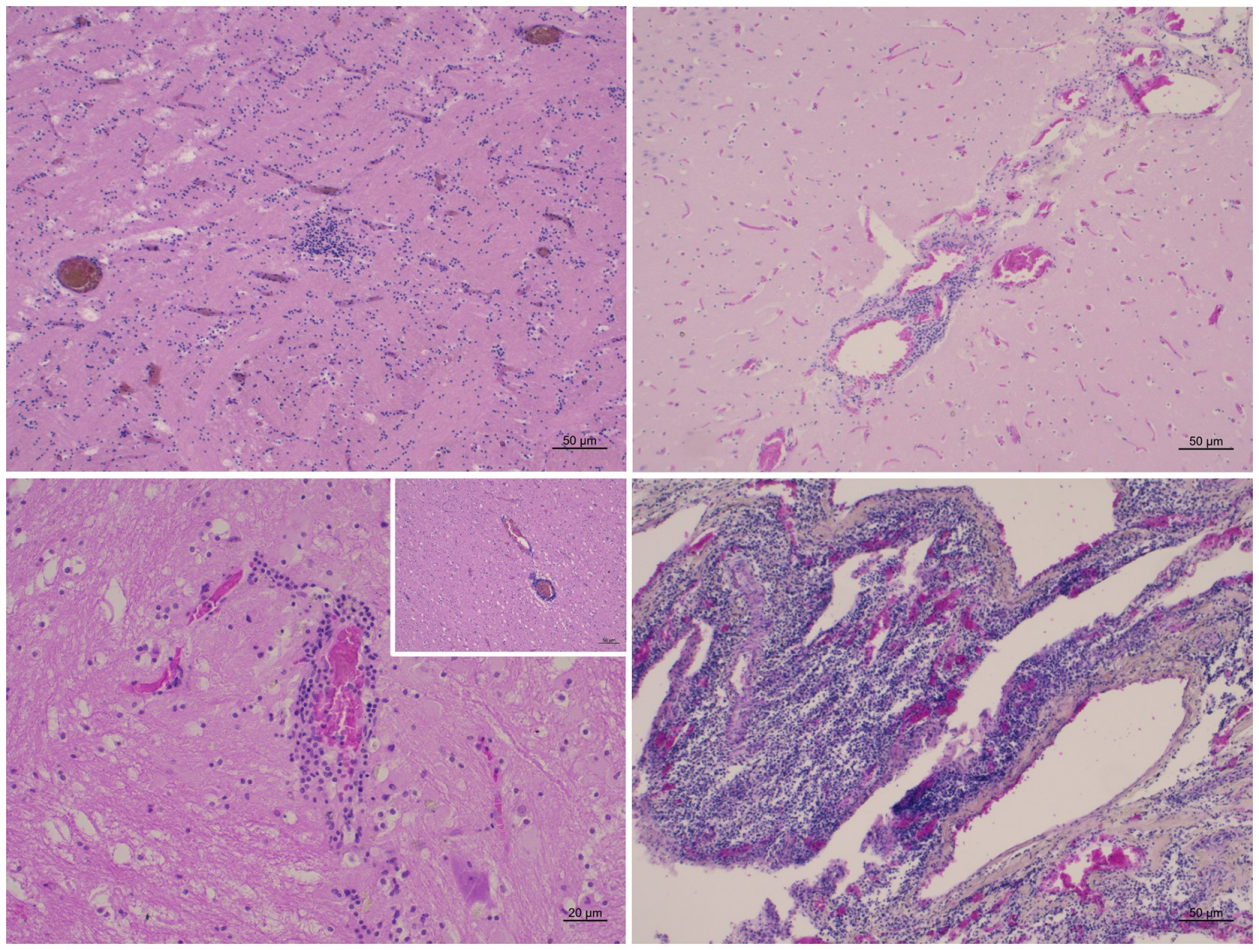
Neuropathological changes in stranded cetaceans affected by Herpesvirus. Animals are identified using the case identification provided in **Table 1** (ID). (A) Brain cortex (ID10). Focus of gliosis. HE. (B) Occipital Cortex (ID12). Mild non suppurative meningitis. HE. (C) Thalamus (ID12). Lymphoplasmacytic perivascular cuff. Inset: Parietal cortex. Lymphoplasmacytic perivascular cuffs. HE. (D) Plexus choroideus (ID 12). Severe and diffuse non-suppurative plexus choroiditis. HE.

Despite the detection of HV infection in the CNS of six dolphins (three AHV and three GHV), all of which were male striped dolphins, neuropathological characterization was only feasible for five of them (ID 2, 6, 7, 10, 12) (**Figs 2** and **3**). HV-associated lesions are described in **Table 3** and included three mild meningitis with minimal and mild perivascular cuffing (**Fig 2B**), mainly composed of lymphocytes and plasma cells. Astro-microgliosis was present in all five individuals, being mild in individuals ID 2, 6, 7 and 12, while severe astro-microgliosis was evident in individual ID 10. In addition, cases ID 6 and 10 exhibited severe neuronal necrosis accompanied by neuronophagia (**Fig 2A**). Furthermore, animal ID 12 displayed minimal neuronal necrosis and minimal malacia, whereas case ID 7 showed mild malacia. Coinfections at the cerebral level were detected in three individuals (ID 2, 7, and 12), primarily with CeMV (ID 2 and 12), *Toxoplasma gondii* (ID 7 and 12), and *Brucella ceti* (ID 12). In the case of ID 6, no cerebral coinfections were identified, but CeMV was identified in several tissues, including pharyngeal tonsils, prescapular, tracheobronchial and pulmonary lymph nodes and urinary bladder. Additionally, *Salmonella* Tsevie was detected in prescapular lymph node, and *Photobacterium damselae* in the liver and spleen. In the case of ID 10, no cerebral coinfections were detected, but CeMV was detected in the lung and heart. Unfortunately, no included bodies were observed in any of the animals investigated.

**Table 3:** Neuropathology in HV-positive dolphins. Abbreviations: ID, case identification; Ref. lab, Laboratorial reference; SF, Subfamily of Herpesvirus; M, meningitis; PC, perivascular cuffing; Astro-Mg, astro microgliosis; NN, neuronal necrosis; O, oedema; INCIBs, intranuclear and/or intracytoplasmatic inclusion bodies; H, haemorrhage; AHV, Alphaherpesvirus; GHV, Gammaherpesvirus; absent (-); minimal (+); mild (++); moderate (+++); severe (++++); CeMV: Cetacean Morbillivirus.

ID	Ref. lab	SF	Cerebral cortex								Lesions in other regions	Associated lesions	Co-infections	References
			M	PC	Astro-Mg	Malacia	NN	O	INCIBs	H				
2	95842	GHV	-	-	++	-	-	++	-	-	Mild non-suppurative plexus choroiditis	Spongiosis	CeMV	[42, 66, 67]
6	5386	GHV	++	++	++	-	+++	+	-	++	Diffuse mild non-suppurative encephalitis and neuronophagia. Haemorrages in cervical spinal cord	-	-	-
7	62728	GHV	++	+	++	++	-	+	-	-	-	-	*Toxoplasma gondii*	[42, 70]
10	12046	AHV	-	-	++++	-	++++	+	-	+	Mild pyogranolomatous plexus choroiditis with scattered syncytia, non-suppurative perivascular cuffing in pons	-	-	-
12	24621	AHV	++	++	++	+	+	+	-	+	Severe non-suppurative plexus choroiditis and encephalitis in thalamus	Protozoan cysts and granulomatous encephalitis, spongiosis	CeMV, *Toxoplasma gondii*, *Brucella ceti*	[70]

## Discussion

Cetacean populations are widely affected by HV, with the highest reported prevalence found in the Mediterranean Sea [15]. Despite its widespread presence, the actual impact of this virus on cetacean populations remains largely unknown. In this study, we describe the epidemiological characteristics of infected individuals, conduct a phylogenetic analysis of the obtained sequences, and evaluate the potential pathological lesions, with a particular focus on the associated neuropathology. This comprehensive approach enhances our understanding of HV in odontocetes from both epidemiological and pathological perspectives, thereby providing better insight into its impact on these populations.

Of the 18 HV-positive animals, the majority were males (66.7%, 12/18), while females constituted a smaller proportion (27.8%, 5/18). In one case (5.5%, 1/18), the sex of the animal could not be determined due to advanced decomposition (**Table 1**). The disparity in HV infection based on sex has been previously noted in Spain (Cantabria [14] and the Canary Islands [29]), the Netherlands [18], Brazil [5], and Japan [6], with a higher presence of HV in males than females. However, in other studies, no sex predisposition was found [15–17].

In our study males contained a greater number of positive tissues (average: 2.16, 26/12) than females (average: 1.6, 45/20). Four out of the 12 positive males (33.4%) had three or more positive tissues, whereas only one out of five positive females (20.0%) showed this scenario. Similarly, Sierra *et al*. reported the virus in multiple organs, ranging from two to six, in five of the eight males, whereas none of the positive females (0/4) showed this condition, indicating that positive females had the infection in only one tissue [29]. Bento *et al*. observed a higher proportion of animals with more than one positive tissue in males (5/7) than in females (3/7), although the maximum number of tissues with virus presence per individual was higher in females, with five (n = 1) and four (n = 2) tissues, compared to a maximum of three (n = 2) in males [17]. Vargas-Castro *et al*. found that the two HV-positive females reported had only one positive tissue, while in males, the number of tissues with virus presence ranged from two to seven [14]. In contrast, another study reported that positive females had a higher number of positive tissues, averaging 4.37 (70/16), compared to positive males, who averaged 2.25 (45/20). Additionally, half (8/16) of the positive females had more than three positive tissues, whereas this was observed in only three of the 20 positive males [15]. In line with this, Felipe-Jiménez *et al*. reported that HV-positive females had between 1 and 4 tissues with virus presence, and in 60% of the cases (3/5), the virus was detected in more than one tissue. In contrast, positive males had the virus in one (n = 1) or two (n = 2) tissues [16].

The underlying cause of this heterogenicity has yet to be clarified, and as previously suggested, further research is needed to determine if sex could be considered a risk factor in HV infection [15, 29].

In terms of age distribution, among the infected individuals, ten were classified as adults, while eight fell into the juveniles-subadults category (**Table 1**). In this study, no infected calves or neonates were found. Our findings align with a study on franciscanas (*Pontoporia blainvillei*) in Brazil, where adults showed the highest prevalence of HV infection [10]. Similarly, in some studies conducted on beaked whales [16] and striped dolphins in Spain [14], all HV-infected individuals were adults. However, other studies have found the lowest or among the lowest prevalence rates in this age group [17, 29]. The presence of HV infection in adults could be explained by the ability of HV to establish lifelong latent infections [44, 45]. Nonetheless, these results should be interpreted with caution, as the mentioned studies discuss prevalence based on age groups, while our study reports absolute numbers rather than proportions. Regarding the host species, although HV infection has been documented in three mysticete species in the Mediterranean Sea [9, 33], our study identified infection in two odontocete species: striped dolphins (n = 15) and bottlenose dolphins (n = 3) (**Table 1**). These species have previously been reported as common HV hosts both in the Mediterranean Sea [13, 15, 38, 41, 71] and other regions worldwide [14, 17, 27, 29, 36, 37].

In our study, we acknowledge the inherent limitations associated with the small sample size, particularly regarding comparisons between groups, such as age or sex, which may not be strongly supported by the available data. Additionally, the opportunistic nature of sampling stranded animals, which often results in a non-representative sample of the broader population, presents challenges in extrapolating prevalence rates. While these constraints are common in studies of this nature, they limit the generalizability of our findings and should be considered when interpreting the results.

In this study, the highest positivity rates were obtained in the genital, nervous, and integumentary systems, in descending order. These findings corroborate previously proposed results regarding HV tropism in these three organ systems in odontocetes [15]. However, several sex- and age-related differences were observed regarding tissue tropism. In females, HV was most frequently detected in the lymph nodes and lungs (n = 3), while in males, it was predominantly found in the lymph nodes (n = 7) and CNS (n = 6). However, when examining proportions, HV showed a higher tropism for the lungs (75%, 3/4) and skin (66.7%, 2/3) in females, whereas in males, it was more prevalent in the CNS (66.7%, 6/9) and kidneys (66.7%, 2/3). While there was a 100% detection rate of HV in the liver, genital mucosa, and lingual mucosa in males, it should be noted that this finding is limited to just one sample per tissue. Our findings are consistent with those reported by Bento *et al*., where the primary organ showing molecular positivity for HV in males was the kidney, whereas in females it was the lung [17]. Additionally, we identified three positive liver samples and the only genital sample with molecular positivity in males [17]. However, these results differ from other studies. For example, Vargas-Castro *et al*. (2021) found a greater proportion of HV-positive CNS and genital mucosa samples in females than in males [15]. Furthermore, Felipe-Jiménez *et al*. (2021) identified lung and kidney as the main HV-positive samples in males and females, respectively, which is the opposite of what we observed in our study [16].

On the other hand, with regard to age, HV was most frequently detected in the lymph nodes (n = 5) and CNS (n = 4) in adults, whereas in juveniles, it was primarily found in the lymph nodes (n = 6) and lungs (n = 5). However, when considering the proportion of positive samples relative to the number of samples analyzed, the highest positivity rates in adults were observed in the skin (66.7%, 2/3) and CNS (50%, 4/8). In juveniles, the highest positivity rates were found in the lungs (71.4%, 5/7) and lymph nodes (40%, 6/15). Some of these results are similar to those obtained in another study on HV tropism in Mediterranean Sea odontocetes, which also found that the skin was more frequently infected in adults, with the CNS being the second most frequently infected tissue [15]. However, HV infections in the hematopoietic system were uncommon [15], whereas our results indicate that the lymph nodes were the second most infected tissue in this age group. In another study conducted in Spain (Canary Islands), the most commonly infected tissues in adults were the kidneys and lungs [16]. Meanwhile, in Portugal and Spain (Galicia), the only positive CNS samples were found in juvenile individuals. In this age group, the lungs and lymph nodes were the third most common tissues with positive samples [17].

These discrepancies suggest that more studies are needed to better understand the factors influencing HV tissue tropism in cetaceans, such as sex and age.

**Fig 4:**
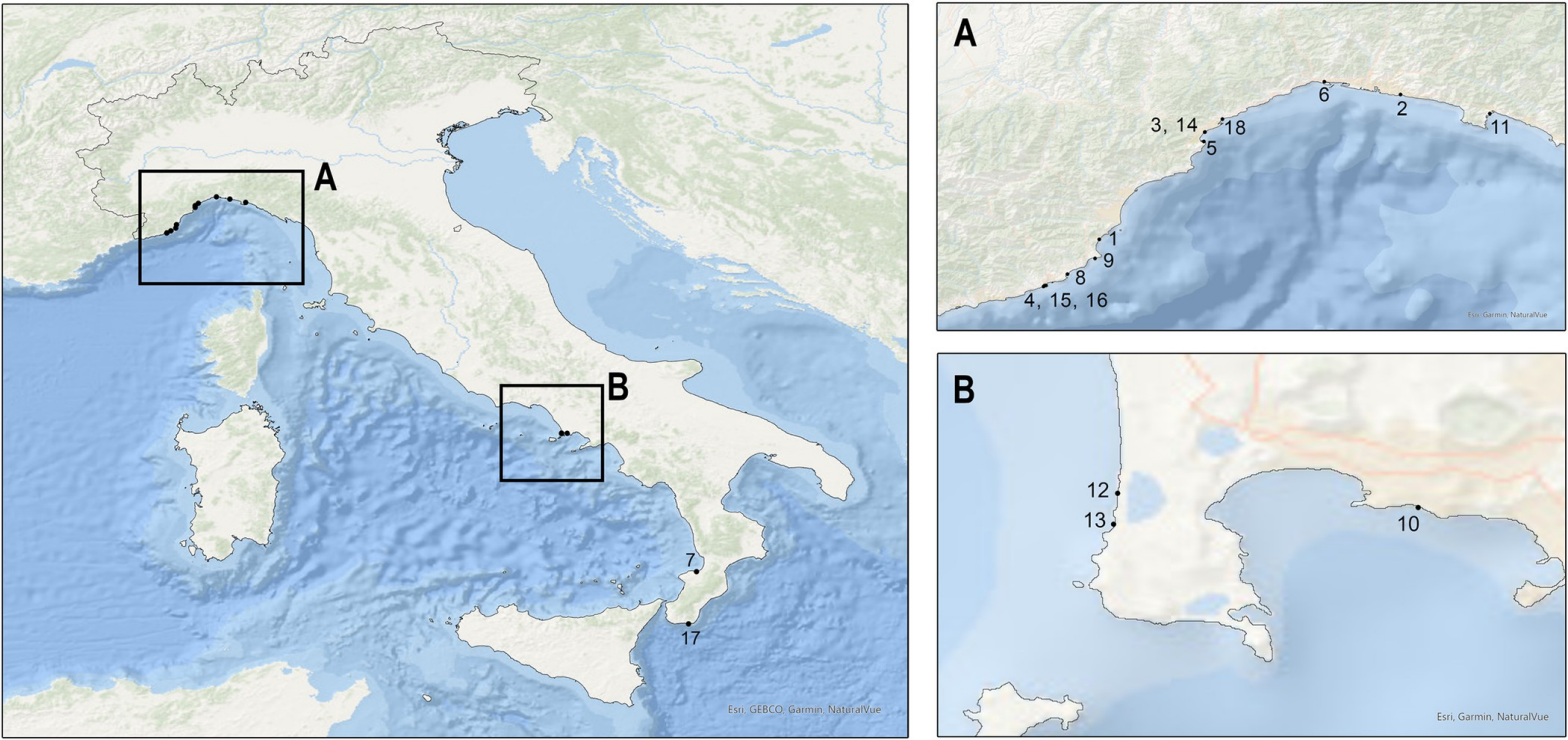
Stranding sites of cases 1–14. Geographical mapping was created with ArcGIS Pro software (versión 3.1) using the geographical coordinates found from the strandings. Animals are identified using the case identification provided in **Table 1** (ID).

Regarding the geographical locations of the strandings of affected dolphins, 13 were found in Liguria, three in Campania, and two in Calabria regions (**Fig 4**). In the Ligurian Sea, high levels of exposure to various pathogens, including HV, have been previously detected and correlated with concerning levels of organochlorine pollutants [71]. A recent study identified Calabria and Campania as the regions with the highest number of CeMV-positive cetaceans, and it was hypothesized that there is a link between these infections and environmental factors, such as high levels of chemical pollutants [69]. Overall, these results may support the hypothesis that HV is an important factor to consider when studying cetaceans as marine ecosystem sentinels, as previously proposed [15].

The phylogenetic analysis unveiled a higher occurrence of GHV sequences (n = 11) compared to AHV (n = 8) (**Fig 1**). Moreover, GHV sequences clustered into two distinct groups, whereas AHV sequences exhibited a broader dispersion within the phylogenetic branch corresponding to this viral group, suggesting a greater genetic diversity within AHV. Both findings are in accordance with what has been previously established for herpesviruses in Spanish Mediterranean and Brazil cetacean populations [15, 19]. Nevertheless, there are discrepancies when comparing these results to those obtained in different global regions. A study involving stranded cetaceans in Spain (Galicia) and Portugal revealed a higher presence and greater genetic variation of AHV compared to GHV [17]. Conversely, in riverine dolphins from Brazil, AHV was identified in only one individual (n = 1), while three different GHV sequences were found in a larger number of individuals (n = 20) [10]. Additionally, in the Canary Islands (Spain), only AHV sequences (n = 10) were detected in stranded beaked whales (n = 8) [16]. These discrepancies could indicate variations in HV circulation across different geographical regions and/or among the host species involved. A meta-analysis study would be helpful to shed light on these aspects in a global perspective.

Another notable finding from the phylogenetic study is that some GHV sequences clustered together with a sequence detected in a striped dolphin stranded on the Spanish Mediterranean Coast (MH521298) and another sequence found in a bat species (MF579868). This outcome aligns with the preliminary BLAST analysis conducted on the sequences described in this study, which exhibited the highest identity with these two previously documented sequences. Such findings may indicate the existence of a distinct group of GHV capable of infecting cetaceans, potentially highlighting a novel evolutionary relationship within this viral subfamily. Interestingly, while the sequence from the bat species also falls within this cluster, it demonstrates greater genetic divergence. Further research is needed to understand the implications of these findings and to elucidate the mechanisms underlying the clustering of HV sequences from dolphins and bats.

Case 13 demonstrated simultaneous infection by two distinct GHV strains in different organs (skin and lungs). The occurrence of multiple herpesvirus strains infecting the same individual has been observed in various cetacean species [3, 5, 10, 13–17, 28], suggesting it is a common feature in HV infection among these animals.

Histopathology was not possible in five of the 18 individuals due to an advanced state of autolysis. Only three animals showed potentially HV-related lesions affecting vital organs, primarily resulting in non-suppurative meningoencephalitis (ID 6, 7, and 12) and lymphoplasmacytic nephritis (ID 12). However, in the studied animals, the main lesions suggestive of HV involvement consisted of lymphoid depletion and inflammation of the affected tissues. On one hand, central nervous system (CNS) damage was evident across all studied cases, as detailed in **Table 3** and **Figs 2** and **3**. Astro-microgliosis, observed in all five individuals, ranged from mild in cases 2, 6, 7, and 12, to severe in individual 10. Remarkably, the severity of astro-microgliosis in case 10 correlated with the absence of co-infections with other agents at the CNS level. This animal and case 6 exhibit moderate and severe neuronal necrosis, respectively, accompanied by neuronophagia (**Fig 2A** and **2C**). Additionally, three cases presented mild meningitis with minimal to mild perivascular cuffing (**Figs 2B**, **3B** and **3C**), primarily composed of lymphocytes and plasma cells. Animal 12 exhibited minimal neuronal necrosis and minimal malacia, while case 7 showed mild malacia (**Fig 2D**). Unfortunately, no INCIBs were detected in any case.

These findings align with a previous study characterizing CNS damage associated with HV, which identified primary neuropathological features including perivascular cuffing, meningitis, microgliosis, neuronal necrosis with focal neuronophagic nodules, malacia, hemorrhage, and choroiditis [29]. This is particularly interesting in cases ID 6 and 10, that did not present any coinfection in the CNS, which would indicate that the reported lesions were associated exclusively with HV. The detection of a GHV infection in case 6 would confirm the potential of this HV subfamily to induce neurological damage, while excluding the involvement of other potential neurotropic agents in cetaceans. Previous reports have noted the presence of GHV associated with CNS lesions, although these lesions were often mild or nonspecific [15, 29, 42]. Some cases showed coinfections with AHV or CeMV [29, 42], while other studies were unable to determine the presence of all potential neurotropic agents [14, 15].

In another study, mild malacia was more frequently detected in HV-positive animals, while it was mainly minimal in animals infected by *T*. *gondii*, *Brucella* spp., and CeMV [43]. Accordingly, case 7 showed a coinfection with *Brucella ceti*, *T*. *gondii* and CeMV. Interestingly, syncytia, a characteristic feature of CeMV [42, 43], were observed in the choroid plexus of case 10, which tested molecularly positive for CeMV (**Table 1**) in the lungs and heart despite the absence of co-infection in the CNS. Similarly, previous studies have detected lesions consistent with CeMV, such as chronic interstitial pneumonia and lymphoplasmacytic cystitis with intracytoplasmic inclusion bodies, in some HV-infected tissues of coinfected animals, despite negative molecular diagnosis for CeMV [15]. Moreover, protozoan cysts and granulomatous encephalitis attributed to *T*. *gondii* [43] were present in case 12.

Another particular aspect was the detection of plexus choroiditis ranging from mild to severe in three individuals (**Figs 2E and 3D**). This feature was previously described in CNS HV infection in cetaceans [43], and it may suggest that the choroid plexus could serve as a route of entry.

To better characterize neuropathological lesions and changes associated with HV, it would be highly beneficial to implement immunohistochemistry for this pathogen [29], to identify and localize the antigen in infected tissues. Additionally, double labeling with indirect immunofluorescence would be useful to differentiate lesions associated with various neurotropic pathogens in cases of coinfections, as the lesions and changes often overlap among the involved agents. Such advancements are thus crucial not only for understanding HV-related pathology but also for managing cases involving co-infections with other neurotropic agents.

On the other hand, case 12 showed an infection along with a HV with concomitant lymphoplasmacytic nephritis (**Table 2**). HV-associated nephritis has been previously reported in odontocetes, mainly in beaked whales [16, 30] but also in striped dolphins [15], as identified in this study.

**Fig 5:**
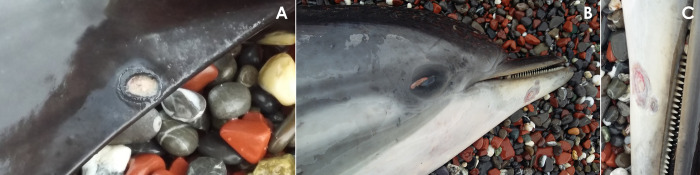
Gross necropsy findings in the skin of case 2 (ID 2). A) Focal, well-delimited, round-shaped, white, cutaneous ulceration affecting the skin of the peduncle, B) focal, well-delimited, round-shaped, yellowish to red, epidermal erosion affecting the skin of the mandibular rostrum, C) detailed view of the erosive lesion located on the rostrum.

**Fig 6:**
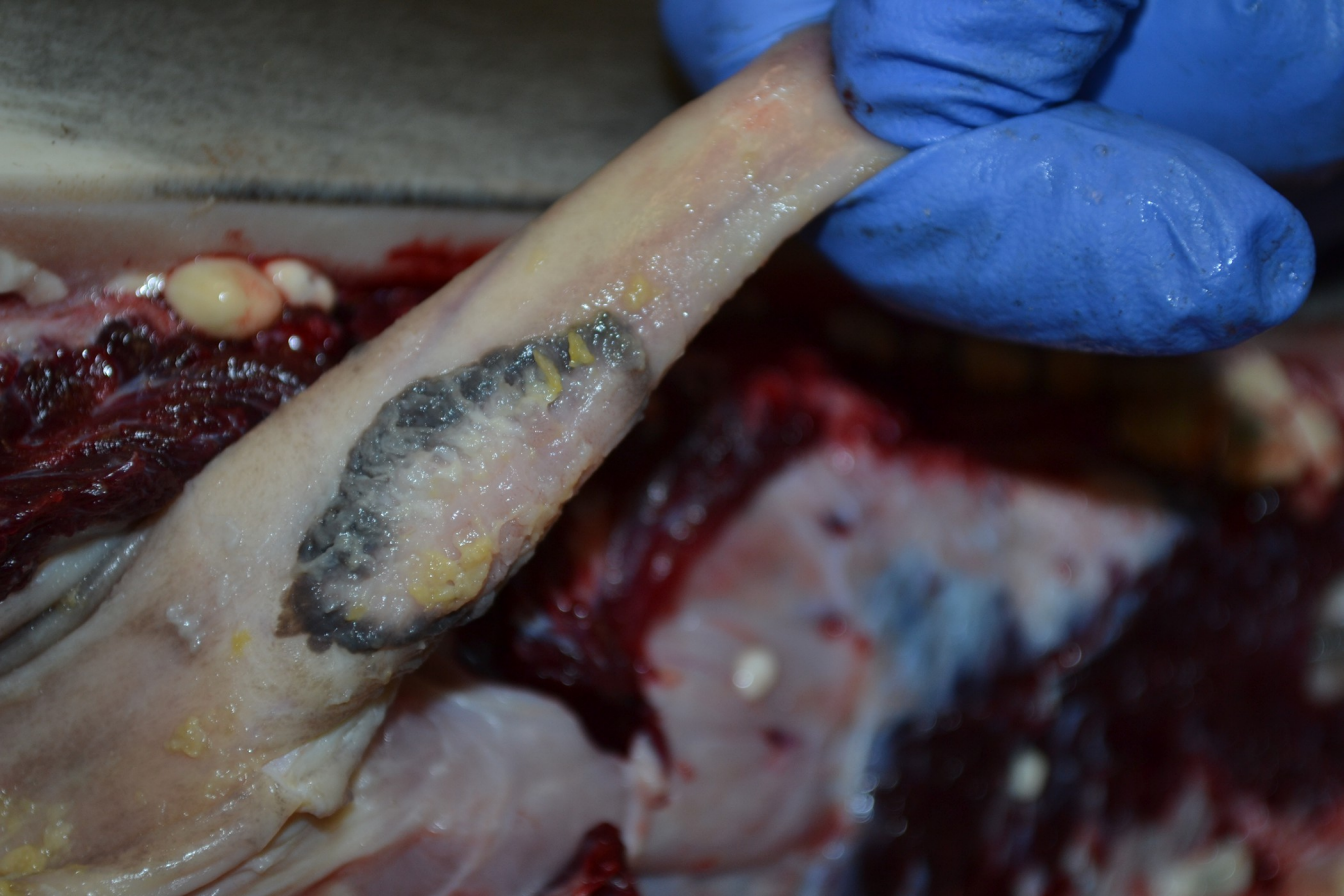
Gross necropsy finding in the male reproductive system of case 6 (ID 6). Focal, 2 cm in diameter, well-delimited, ovoid-shaped, whitish and peripherally pigmented, proliferative balanitis, with multifocal yellowish caseous exudate, located on the middle of the penis.

**Fig 7:**
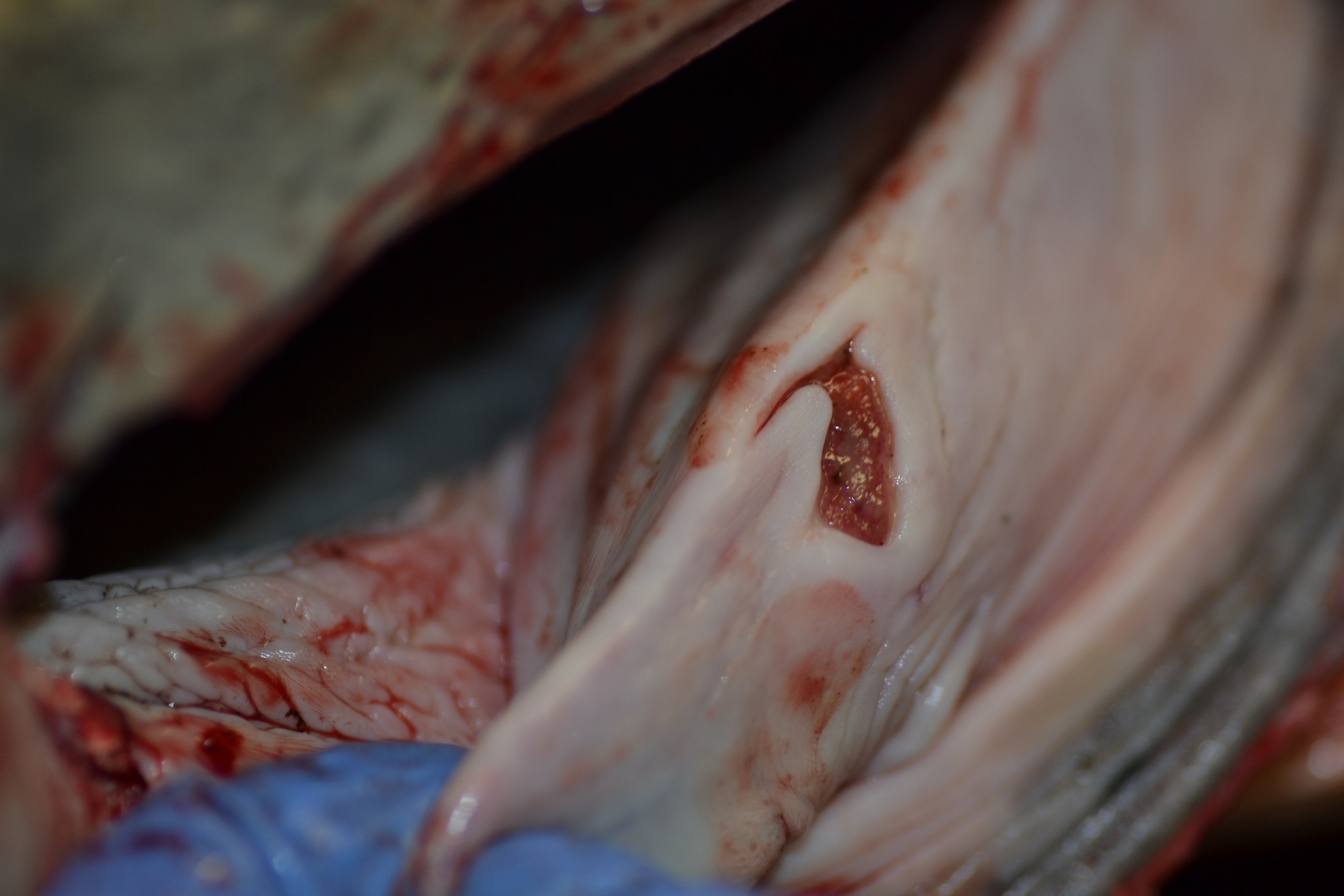
Gross necropsy findings in the upper digestive tract of case 6 (ID 6). Focal, 2–3 cm in diameter, well-delimited, irregular-shaped, reddish epidermal ulcerative lesion affecting the mucosa of the esophagus, around the larynx.

The other lesions potentially related to HV were found in non-vital tissues such as skin (**Fig 5**), genital mucosa (**Fig 6**), and lingual mucosa (**Fig 7**).

In our study, we identified two distinct skin lesions. Animal 2 exhibited multifocal skin erosions in the rostrum, left flank, and peduncle (**Fig 5**), which microscopically corresponded to degenerative and necrotizing dermatitis. These lesions presented areas of epithelial erosion and necrosis, accompanied by mononuclear inflammatory infiltrate. Conversely, animal 14 showed severe pyogranulomatous panniculitis. In cetaceans, a wide range of skin lesions associated with HV has been described [5, 8, 15, 31–33, 77–79]. Among them, epidermal necrosis [77, 78] and panniculitis [5] have been previously considered consistent with HV infections. However, pyogranulomatous panniculitis in marine mammals has been more commonly associated with *Mycobacterium marinum* [80, 81]. It should be noted that mycobacteria were not specifically screened in this study. Therefore, the possibility of co-infection with this agent cannot be ruled out.

In our study, a case of hyperplastic balanitis (ID 6) was identified at the genital level (**Fig 6**). Additionally, ballooning degeneration of epithelial cells at the level of the stratum corneum was detected. This observation aligns with previous findings regarding epithelial hyperplasia as a common feature associated with HV in the genital mucosa of cetaceans [8, 11, 15, 18, 35–37]. Furthermore, hydropic degeneration of epithelial cells has been previously documented in other genital lesions linked to HV in odontocetes [7, 36].

This animal also presented an ulcerative glossitis and esophagitis. Histopathologically, the lingual ulcer (**Fig 7**) consisted of glossitis with epithelial hyperplasia. In recent years, there has been an increase in the identification of upper digestive tract lesions (including oral, lingual, pharyngeal and esophageal) associated with HV in cetaceans [3, 5, 7, 15, 41, 82]. The clinical importance of these findings and the pathogenic and transmission routes remain unknown [41]. It has been proposed that oro-genital contact during suckling or socio-sexual behaviors, such as ’goosing’, may be implicated in this transmission [41], as some of the sequences present in these lesions share significant identity with several genital sequences, raising questions about their origin [15, 41]. Surprisingly, in a previously described case, a Risso’s dolphin (*Grampus griseus*) presented lesions associated with AHV and GHV in the genital and esophageal mucosa, respectively, with both infections associated with similar lesions [15]. In the case described in this article, the animal also presented with lesions at the lingual and genital levels, both associated with molecular positivity to HV.

Coinfections were detected in 83.3% (15/18) of the studied animals (Table 1), involving bacteria (n = 12), viruses (n = 11), protozoa (n = 3), helminths (n = 3), and/or fungi (n = 2). Interestingly, the predominant agent identified in these coinfections was CeMV (n = 11). Given that HV is known to induce immunosuppression in cetaceans [4], it is common to observe concomitant infections with other agents, particularly CeMV [13, 15, 17, 19, 29, 41–43].

**Fig 8:**
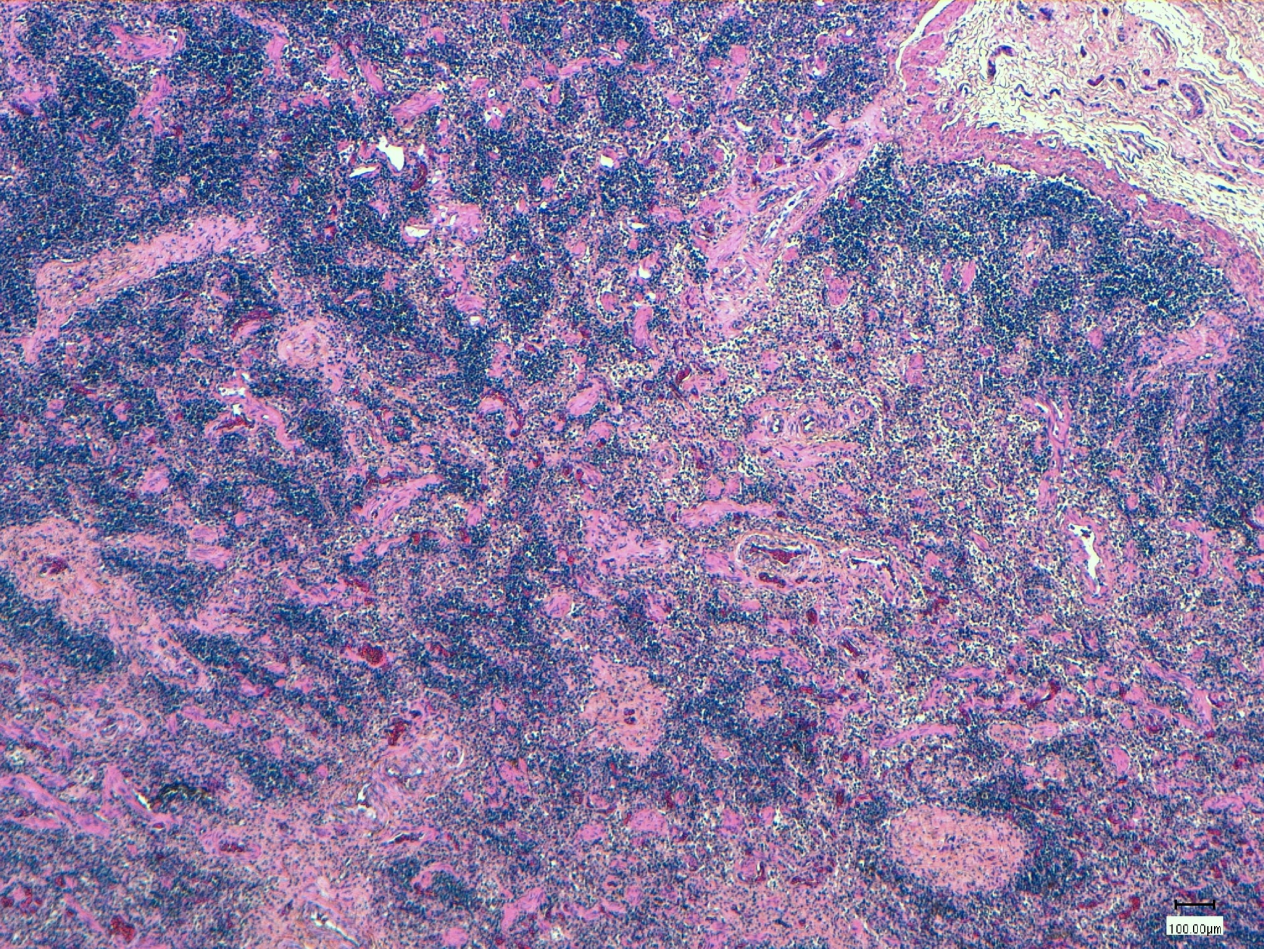
Histopathological findings in lymph nodes in case 3 (ID 3). Overall decrease in the number of lymphoid cells associated with the periarteriolar lymphoid sheath and follicles.

Linking these findings to the observed pathology in the hematopoietic system, it is notable that four cases showed lymphoid depletion (**Fig 8**): three of them at the level of lymph nodes (ID 3, 6, 15) and one at the splenic level (ID 11) (Table 2). Notably, the three cases with lymphoid depletion in the lymph nodes presented coinfections with other agents, while the case with splenic lymphoid depletion did not exhibit any coinfections. This underscores the complex interplay between viral infections and the immune response in cetaceans, highlighting the need for further investigation into the mechanisms underlying these interactions.

This study contributes to enhance our understanding about HV infections in odontocetes, particularly in the Mediterranean Sea by providing comprehensive insights into the epidemiology, pathology, and molecular characteristics of HV. However, it also underscores the need for further investigation to fill existing knowledge gaps.

## Conclusions

In conclusion, this study significantly advances our understanding of HV infections in odontocetes, with a particular focus on the Mediterranean Sea, by shedding light on their epidemiological and pathological aspects. Our findings reveal notable patterns, including a higher presence of HV in males and a greater number of positive tissues in males compared to females. Additionally, a predominance of HV infection in adults was observed. The phylogenetic analysis uncovers a higher occurrence of GHV sequences and greater genetic diversity within AHV. Furthermore, the lesions potentially related to HV, characterized by lymphoid depletion and tissue inflammation, underscore the pathological significance of these infections. Regarding the neuropathology, astro-microgliosis was observed in all five studied individuals, with three showing mild meningitis characterized by minimal to mild perivascular cuffing, and two cases presenting severe neuronal necrosis and neuronophagia. Importantly, the documentation of concurrent infections with other pathogens, particularly CeMV, highlights the complex nature of infectious diseases in cetaceans. However, the presence of lesions at the CNS with molecular positivity for GHV, but not for other neurotropic agents, would confirm the potential of this HV subfamily to induce neurological damage. These results emphasize the importance of ongoing research to elucidate the factors driving HV infections in odontocetes and their broader impacts on population health and conservation efforts.

## Supporting information

S1 Checklist Inclusivity in global research. (DOCX)

## List of abbreviations

HV: Herpesviruses
CReDiMa: Italian National Reference Centre for Diagnostic Investigations in Stranded Marine Mammals
AHV: Alphaherpesviruse
GHV: : Gammaherpesvirus
CNS: Central Nervous System
IZS: Istituti Zooprofilattici Sperimentali
DCC: Decomposition Condition
CeMV: Cetacean Morbillivirus
DNApol: DNA polymerase
COD: Cause of death
INCIBs: Intranuclear and/or intracytoplasmatic inclusion bodies
